# Social care and changes in occupational accidents and diseases - the situation in Eastern Europe in general and for skin diseases in particular

**DOI:** 10.1186/1745-6673-4-28

**Published:** 2009-11-18

**Authors:** Kathrin R von Hirschberg, Björn Kähler, Albert Nienhaus

**Affiliations:** 1 Institution for Statutory Accident Insurance and Prevention in the Health and Welfare Services, Hamburg, Germany

## Abstract

**Background:**

As a consequence of the disintegration of the state systems and the expansion of the European Union, there have been marked changes in the political and social affiliations of the countries of Eastern Europe. Of the 22 countries in Northeastern, Centraleastern, Southeastern and Eastern Europe, 12 are now members and 10 are "new" neighbours of the European Union. The accident insurance systems and changes in occupational accidents and occupational diseases in eastern European countries are described. Changes since EU and visible differences from non-EU countries are analysed. Special emphasis is given to occupational skin diseases.

**Methods:**

The available data from the European Union (MISSOC and MISSCEEC Studies on the Social Protection Systems), the database "Social Security Worldwide" (SSW) of the International Social Security Association (ISSA), the International Labour Office Database (LABORSTA), the World Health Organization (WHO) and the annual statistical reports of the different countries were analysed with respect to changes in occupational accidents and occupational diseases. To find missing data, 128 ministries and authorities in the 22 countries in eastern Europe were researched and 165 persons contacted.

**Results:**

The social insurance systems were very different in the different countries and some were better established than others. Moreover, not all data were available. For these reasons, detailed comparison was not always possible. The occupational accident rates are decreasing in more than half the countries. In contrast, the fatal accident rates have increased in half the countries. The number of newly registered occupational diseases is decreasing in more than half the countries. The rates for occupational skin diseases in 2006 were particularly high in the Czech Republic, Poland and Slovakia. In half the countries (four out of eight), the number of occupational skin diseases is decreasing. A reliable analysis of any correlation between EU membership and the rates of occupational accidents and occupational diseases was not possible, because of missing current data.

**Conclusion:**

Comparison of the social insurance systems and changes in occupational accidents and occupational diseases in 22 countries in eastern Europe makes it clear that further effort is needed to develop registration and notification procedures. Only then will it be possible to analyse changes, to map successes and problems and perhaps to initiate necessary improvements. Standardisation of the documents must also be improved, to allow international comparisons between the systems.

## Background

As a consequence of the disintegration of the state systems and the expansion of the European Union, there have been marked changes in the political and social affiliations of the countries of Eastern Europe. Of the 22 countries in Northeast, Centraleastern, Southeast and Eastern Europe, 12 are now members and 10 are "new" neighbours of the European Union. There have been extensive social and political reforms in the new members of the Union, as our new neighbours approach European "conditions", and this enhances the interest in a detailed analysis of the situation. For the present study, it was particularly interesting to look at the areas of the social security systems and the occupational safety and health system. The focus was on a comparative consideration of social security and on the changes in the rates of occupational accidents and occupational diseases. It was also investigated whether there had been changes in this context since entry to the EU and whether there are differences compared to non-EU countries. Special emphasis was given to the analysis of occupational skin diseases in eastern European countries.

## Methods

Currently available data on the issues at point were collected, in particular, the compilations of the European Union (MISSOC and MISSCEEC Studies on the Social Protection Systems) and the database "Social Security Worldwide" (SSW) of the International Social Security Association (ISSA) on the social insurance systems in eastern European countries. There was little available information for the eastern European countries which are not members of the EU.

Analysis of the changes in occupational accidents and occupational diseases is based on materials from the International Labour Office Database (LABORSTA), the World Health Organisation (WHO) and -particularly for the non-EU member countries - on direct contact with institutions and persons in these countries. For useful literature and internet sites see additional file 1. To provide missing data, a total of 128 ministries, authorities, statistical offices and institutions in the 22 countries of Eastern Europe were researched; 165 possible contacts were localised and sent letters, enquiring about current data and the legal social insurance system for dealing with occupational accidents and occupational diseases. In all, 39 persons responded to this, corresponding to a response rate of about 23% (Table 1).

**Table 1:** Research of Contacts in ministries, authorities, agencies

Country	Researched Ministeries/Institutions/Authorities	Contacts	Responses
**Higher order**	ILO - Unit Health Care BAUA European Association for Communication in Health Care DSVEV Center of European Policy Studies WHO - Health Information Unit International Commission of Occupational Health	15	5

**Albania**	Ministry of Health Albanian Epidemiological Association State Labour Inspectorate Social Insurance Institute	4	1

**Belarus**	Ministry of Health Ministry of Labour Ministry of Statistics	3	0

**Bosnia-Herzegovina**	Ministry of Health Federation of Bosnia and Herzegovina Ministry of Health and Social Protection of Rep. Srpska Society of Social Medicine Agency for Statistics of Bosnia and Herzegovina Office of Statistics	5	0

**Bulgaria**	Bulgarian Public Health Association National Centre of Public Health Protection National Expert Medical Commission Clinic for Occupational Diseases Association "Workplace Health and Safety Promotion" National Center of Health Informatics NCO Bulgaria National Center of Hygiene, Medical Ecology National Health Insurance Fund Ministry of Labour and Social Policy-General Labour Inspectorate Ministry of Health	12	3

**Croatia**	Ministry of Health State Secretary for Health State Secretary for Social Welfare Croatian Society on Occupational Health Croatian National Institute of Public Health Croatian Public Health Association State Inspectorate - Labour Inspection Central Bureau of Statistics	8	1

**Cyprus**	Ministry of Health Cyprus Institute for the Environment and Public Health Cyprus Safety and Health Agency Department of Labour Inspection	4	0

**Czech Republic**	Ministry of Health Institute of Health Policy and Economics National Institute for Public Health Department of Occupational disease Czech Society of Public Health/Health Services WSO International Office for Czech Republic Occupational Safety Research Institute State Labour Inspection Office Dept. of Occupational Medicine	11	2

**Estonia**	National Institute for Health Development Health Protection Inspectorat Estonia Health Insurance Fund Ministry of Social Affairs - Health Care Department	6	1

**Greece**	Ministry of Health and Welfare Ministry of Labour and Social Affairs Hellenic Institute for Occupational Health and Safety ILO Member: Dr. Theodore Bazas National Satistic Service	6	1

**Hungary**	Ministry of Health European Hospital and Healthcare Federation National Institute of Occupational Health Federation of Hungarian Medical Societies Association of Hungarian Medical Societies (MOTESZ) Public Foundation for Research for Occupational Safety National Institute for Strategic Health Research National Health Insurance Fund Administration Department of Labour Protection Hungarian Statistical Office National Center for Public Health	12	2

**Latvia**	Latvian Public Health Association Health Insurance State Agency Health Statistics and Medical technologies State Agency Public Health Agency Institute of Occupational and Environmental Health Ministry of Welfare	7	3

**Lithuania**	Ministry of Health Department of Environmental and Occupational Medicine Kaunas University of Medicine	3	0

**Macedonia**	Ministry of Health National Public Health Institute Macedonian Medical Association Macedonian Occupational Safety Association Ministry of Labour and Social policy State Labour Inspection State Statistical Office	7	2

**Moldavia**	Ministry of Healthcare	1	0

**Montenegro**	Ministry of Health, Labor and Social Welfare Statistical Office of Montenegro	3	0

**Poland**	Ministry of Health Institute of Public Health National Health Fund Nofer Institute of Occupational Medicine National Labour Inspectorate Institute of Occupational Health WSO International Office for Poland	9	2

**Romania**	National Research Institute for Labour Protection Romanian Public Health/Health Management Association ROMTENS Foundation Institute of Public Health Iasi Institute of Public Health Bukarest Romanian Labour Inspectorate	9	3

**Serbia**	Ministry of Health Institute of Occupational Health Serbian Association of Public Health Labour Inspection Ministry of Labour, Employment and Social Policy Statistical Office of the Republic of Serbia Institute of Public Health of Serbia	8	3

**Slovakia**	National Labour Inspectorate Slovak Public Health Association SAVEZ WSO International Office for Slovakia The European Network for Workplace Health Promotion	10	3

**Slovenia**	Ministry of Labour, Family and Social Affairs Ministry of Labour/Inspection Division Department for Health and Safety at Work Ministry of Health of the Republic of Slovenia Slovenian Preventive Medicine Society The European Network for Workplace Health Promotion International Commission on Occupational Health Clinical Institute of Occupational Medicine	10	5

**Turkey**	Ministry of Health Turkish Public Health Association Dokuz Eylùl University	4	0

**Ukraine**	Ministry of Health Ministry of Public Health Center of Medical Statistics Institute of Occupational Health	4	2

**Total**	**128**	**165**	**39**

Changes in occupational accidents and occupational diseases were analysed relative to 2006. If necessary, recourse was made to data for the years 2001-2005.

## Results

### Comparison of the accident insurance systems

In spite of differences in structure, all social insurance systems in the 22 countries considered here share one feature. They all recognise - if sometimes only theoretically - the specific insurance cases of "occupational accident" and "occupational disease". On the other hand, there are marked differences in the way in which this risk is covered according to insurance law. In addition, there are differences in the definition of insurance groups, including the level of the insurance premia and who has to pay these, in the guaranteed payments for total and partial invalidity and in additional payments, such as family and care allowances or pensions for dependents (Table 2 and 3).

**Table 2:** Comparison of the Accident Insurance Systems in the Countries of Northeastern, Central Eastern, Southeastern and Eastern Europe* EU-Members

EU Members	Employment injuries and occupational diseases	Field of application	Special features	Minimum level of invalidity Partial and full invalidity
Bulgaria	Independent component of the compulsory social insurance system	All employees, except for students and persons without a contract of employment. Voluntary insurance for the self-employed and for farmers	Pending reform. Nursing care allowance if occupational invalidity is 90% or more and nursing care is needed. No cumulation with earned income. Cumulation with other pensions is possible (100% of the highest pension plus 50% of other pensions)	Partial invalidity: n.s. Full invalidity: from 50%

Czech Rep.	Independent component of the compulsory social insurance system	All employees; Specific groups: pensioners and students Special system for civil servants	No family allowance; No nursing care allowance; Cumulation with earned income possible Cumulation with other pensions possible Professional rehabilitation.	No compensation. Partial invalidity: from 30% Full invalidity: from 50%

Cyprus	Independent component of the compulsory social insurance system	All employees; self-employed excluded; excluded: employees of the public and diplomatic services of foreign countries, workers on parental farms. Independent agricultural workers aged under 16 years. Voluntary insurance for employees who work abroad.	Family allowance; Nursing care allowance for complete occupational invalidity, requiring nursing care from third parties ca. 45€/p.w. Cumulation with earned income possible. After 1980, Cumulation only possible with widow's pension. Obligation of professional rehabilitation possible European Social Charter since 2000. Ministry of Health and Social Security, Labour Supervision Agencies	Partial invalidity: 10-19% Compensation (Invalidity Compensation) 20-99% Partial Invalidity Full invalidity: 100% plus flat rate/p.w. if nursing care from third parties is necessary

Estonia	No independent insurance. Risks are covered by the health insurance funds (short-term) or pension insurance (long-term).	All employees; No exceptions; No voluntary insurance	No family or nursing care allowance; No cumulation with other pensions; Employee liable in civil law - additional services as compensation, e.g. prostheses, drugs, costs for emergency treatment	Partial invalidity: from 10% compensation Full invalidity: from 40%

Greece	No independent insurance. Risks are covered by sickness, invalidity and dependent insurance.	All employees; No exceptions; No voluntary insurance.	Family allowance: Start of insurance from 1993, no partner allowance, percentage allowance for children Nursing care allowance: Start of insurance from 1993, 25% of the monthly average (1991) of the gross social product; .75% pension payment for occupational invalidity because of psychiatric disease. Cumulation with earned income or other pensions No special rehabilitation measures	No compensation. Partial invalidity: from 50% Full Invalidity: from 80%

Hungary	No independent insurance. Risks are covered by sickness, invalidity and dependent insurance.	All employees: Self-employed, recipients of income support; No voluntary insurance	No family allowance; No nursing care allowance Occupational accident pension: Cumulation with earned income possible. Occupational invalidity pension: cumulation up to 80% of the former income. No cumulation with other pensions. Professional rehabilitation measures up to 50% occupational invalidity. Subsidy for persons providing nursing care	No compensation Partial Invalidity: from 15% to 66% (occupational accident pension) Full Invalidity: from 67% (occupational invalidity pension) 50% for silicosis and asbestosis

Latvia	Independent component of the compulsory social insurance system	All employees; No voluntary insurance	No family allowance; 50% nursing care allowance or nursing care provided 20% reduction if cumulation with old-age pension. Cumulation with earned income possible	Partial invalidity: 10-24%, compensation possible Full invalidity: from 25%

Lithuania	Independent component of the compulsory social insurance system	All employees; Voluntary insurance for self-employed; special systems for the police force, state security, armed forces etc.	No family allowance; No nursing care allowance Full cumulation with other pensions, Cumulation with earned income possible	Partial invalidity: no compensation Full invalidity: from 30%

Poland	Independent component of the compulsory social insurance system	All employees; self-employed No voluntary insurance	No family allowance; Nursing care allowance Choice between cumulation: occupational accident pension 50% plus old-age pension or conversely possible, reduction in pension if additional earned income. Once off payment from employer: ca. 107€ per percentage point of the deterioration in the state of health	Partial invalidity: No compensation; no percentage rate for partial occupational invalidity. Full invalidity: n.s.

Romania	Independent component of the compulsory social insurance system	All employees; Children in full time education; Trainees; Students; Conscripts; Self-employed (voluntary?);	n.s.	Partial invalditiy: from 50%/group III Full invalidity: 100% 3 Groups: I: 100% plus nursing care; II: 100%; III: from 50% restricted employment possible.

Slovakia	Independent component in the compulsory social insurance system	All employees; Students and members of other groups; No voluntary insurance	No family allowance; Compensation of actual nursing care costs; Cumulation with new earned income possible. Reduction if other pension is received.	Partial invalidity: 10%-40% compensation Full invalidity: from 40%

Slovenia	No independent insurance. Risks are covered by sickness, invalidity and dependent insurance.	All employees; Students, trainees, handicapped persons during training, rehabilitation or practical training, persons with second jobs or involved in social activities.	No family allowance; Nursing care allowance; Cumulation with earned income up to the minimum wage is possible. Cumulation possible/Insured persons must decide for a pension. Professional rehabilitation;	Partial invalidity: no compensation Full invalidity: n.s. 3 groups of invalidity: I.: full occupational invalidity; II.: min. 50% occupational invalidity, III.: part-time occupation possible

The information is essentially based on "MISSOC and MISSCEEC Studies on the social protection Systems" of the European Union, the SSW Database (ISSA) (see Appendix) and personal information from the corresponding national authorities.

n.s. not specified

**Table 3:** Comparison of the Accident Insurance Systems in the Countries of Northeastern, Central Eastern, Southeastern and Eastern Europe* Non-EU-Members

Non-EU Members	Employment injuries and occupational diseases	Field of application	Special features	Minimum level of invalidity Partial and full invalidity
Albania	Independent component of the compulsory social insurance system	All employees; trainees, students, self-employed; voluntary insurance possible	Practical problems, as system is being developed. Employees often fail to pay the contributions. No cumulation with other pensions. Professional rehabilitation	Partial invalidity: 10%-33% compensation 33%-66% partial pension Full invalidity: from 67%

Belarus	Independent component of the compulsory social insurance system	All employees; prisoners who work in prison; Excluded: self-employed; Special social insurance for artists, teachers, sportsmen, medical care employees, in public organisations, victims of Tschernobyl.	21% of workplaces in the country are inadequately insured.	Partial invalidity: Group III Full invalidity: 100% 3 Groups: Group I: 100% occupational invalidity plus necessity of treatment; Group II: 100% occupational invalidity; Group III: partial invalidity

Bosnia-Herzegovina	Independent component of compulsory social insurance system.	All employees; self-employed, farmers, employees of religious institutions	No cumulation with other pensions. Special regulations for Republic Srpska and Brcko District.	Partial invalidity: from 20% Full invalidity: from 100%

Croatia	No independent insurance. Risks are covered by sickness, invalidity and dependent insurance.	All employees	Professional rehabilitation, if occupational invalidity at least 50% and aged under 50	Partial invalidity: n.s. Full invalidity: from 51%

Macedonia	No independent insurance Risks are covered by sickness, invalidity and dependent insurance?	n.s.	Because of the lack of financial, institutional and personal resources, the social insurance system is not yet capable of providing functional services. Since 2000 WHO Collaborating Center Skopje: "Specific occupational risks in health care workers- infectious and psychosocial hazards"	Partial invalidity: Group I and II. Full invalidity: 100% 3 Groups: I: Occupational validity can be restored. II: Partial occupational invalidity; III: Complete occupational invalidity

Rep. of Moldavia	No independent insurance? Risks are covered by sickness, invalidity and dependent insurance?	All employees, members of cooperatives, students, trainees, self-employed. Voluntary insurance possible	n.s.	Partial invalidity: Group III Full invalidity: 100% 3 Groups: Group I: Occupational invalidity for all areas of work plus nursing care from third parties; Group II: Occupational invalidity for all areas of work; Group III: Partial invalidity

Montenegro	In development.		Articles 15 and 16 of the Law on Health Care define actions to be taken to protect health at the place of work. Declaration of independence 2006. Restructuring in all areas.	n.s.

Serbia	No independent insurance Risks are covered by sickness, invalidity and dependent insurance	All employees; Self-employed, cooperative members, farmers, artists. Voluntary insurance possible. Special system for members of the armed forces.	Invalidity pension was only introduced in 2008. Declaraton of independence of Kosovo in 02/2008.	Partial invalidity: from 30% Full invalidity: n.s. Eight different invalidity grades

Turkey	No independent insurance Risks are covered by sickness, invalidity and dependent insurance?	All employees, trainees, students, prisoners who work in prison. Special regulations for civil servants, self-employed and farmers. Excluded: part time domestic servants	2004, 9.58% had no social insurance. High additional payment for drugs	Partial invalidity: from 10-25%: compensation Full invalidity: 2/3 = 66%

Ukraine	Individual component of the compulsory social insurance system	All employees; Voluntary insurance possible. Special services for victims of Tschernobyl.	n.s.	Partial invalidity: Group III Full invalidity: 100% 3 Groups: Group I: 100% occupational invalidity plus necessity of treatment; Group II: 100% occupational invalidity; Group III: Partial invalidity

The information is essentially based on "MISSOC and MISSCEEC Studies on the social protection systems" of the European Union, the SSW Database (ISSA) (see Additional file 1) and personal information from the corresponding national authorities.

n.s. not specified

In 12 of the 22 countries, insurance against occupational accidents and occupational diseases is an independent component of the compulsory social insurance system. In nine countries - Estonia, Moldavia, Slovenia, Serbia, Macedonia, Turkey and Hungary -, the risks of occupational accident and occupational disease are covered by health insurance in the short term, pension insurance in the long term and also partially by invalidity and dependent insurance. No statements can yet be made about the social insurance system in Montenegro, which is still being developed.

In the six countries of Macedonia, Moldavia, Romania, Slovenia, the Ukraine and Belarus, three different levels of invalidity are distinguished. Two of these are based on 100% inability to work, with or without long-term need for treatment or medical care. The third level defines partial invalidity. However, as far as is known, this is not clearly defined by a percentage specification of the inability to work. In Moldavia, the inability to work is related to the previous profession. Serbia defines eight levels of invalidity. We were unable to establish precisely how these are differentiated.

In the remaining countries, there are very different minimal rates of loss of workability for receiving partial or full invalidity pensions (Table 2 and 3). In addition, some countries differentiate between partial invalidity payments made as an occupational accident pension (mostly when the rate of loss of workability is low) and a pension for loss of workability, as, for example, in Hungary. In addition, if the rate of loss of workability is low (> = 10%), a onetime compensation payment is made in six countries - Albania, Estonia, Latvia, Slovakia, Turkey and Cyprus -, which replaces the corresponding (minimal) partial invalidity pension. Compensation payments are generally excluded in Greece, Lithuania, Poland, Slovenia, the Czech Republic and Hungary.

The countries with the lowest minimum rates of loss of workability for guaranteeing payment of partial invalidity pensions are Hungary (15%), Bosnia-Herzegovina (20%), Cyprus (20%), Serbia (30%) and Albania (33%). The minimum rate is higher in the other countries.

The minimum rate of loss of workability to obtain full invalidity payments is unusually low in the three Baltic countries. In Latvia, full invalidity payments are paid if the minimum loss of workability is only 25%, with 30% in Lithuania and 40% in Estonia and Slovakia. In contrast, this minimum rate has the comparatively high value of 100% loss of workability in Bosnia-Herzegovina, Macedonia, Moldavia, Romania, the Ukraine, Belarus and Cyprus.

### Comparison and changes in rates of occupational accidents

**Figure 1:**
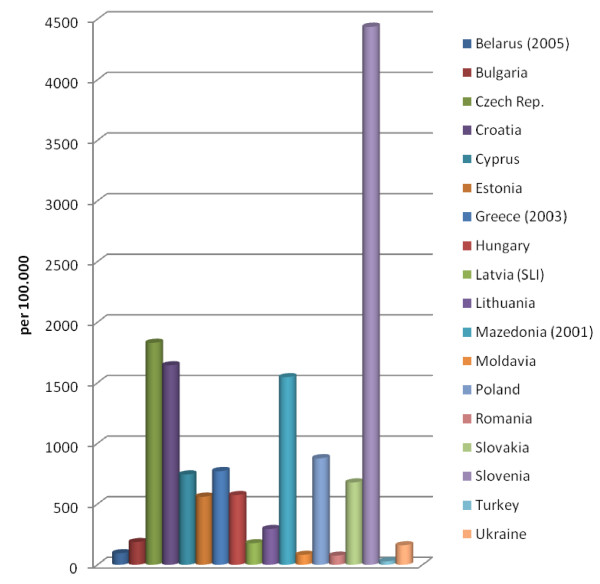
**Non-fatal occupational injuries 2006**. The data provided represent the number of non-fatal occupational injuries per 100.000 workers for the different European countries.

For 2006, data could be determined for 18 of the 22 countries in Eastern Europe. Because of the lack of current data, recourse was made to the data from previous years for Belarus, Greece and Macedonia. For Latvia, recourse was made to the data of the State Labour Inspection (SLI). In 2006, the highest rate of occupational accidents was found in Slovenia. High rates were also found in the Czech Republic, Croatia and Macedonia. On the other hand, the rate was strikingly low in Turkey (Figure 1). The occupational accident rate is not lower in the nine EU countries than in the non-EU countries. It is however striking that the rate of occupational accidents is very low in some of the non-EU countries. As it cannot be assumed that this result can be explained by comprehensive established occupational safety and health guidelines in these countries, it is likely that registration was not performed in a standardised and complete manner. However, the causes could not be conclusively identified.

The change in the accident rate is shown in Table 4. In eleven of the 18 countries for which comparative data were available, the rates of occupational accidents decreased in comparison to the previous year (or the most recent prior year for which data were available):Bulgaria, Greece, Lithuania, Macedonia, Romania, Slovenia, the Czech Republic, the Ukraine, Hungary, Belarus and Cyprus. In contrast, there were increases in Estonia, Latvia, Croatia, Poland, Slovakiaand Turkey. There was little change in Moldavia. The decrease in rate was particularly striking in Bulgaria, Macedonia, Romania, the Czech Republic, Hungary, Belarus, and in the Ukraine (Table 4). There was no evident effect of EU membership on these decreases. Bulgaria and Romania only became EU members in 2007; Belarus and the Ukraine are not EU members. The same applies to the increase in rate, which was found in both EU members and non-EU members.

**Table 4:** Overview Occupational injuries - non-fatal and fatal 2006

EU Members	Occupational injuries (non-fatal) per 100,000	Fatal occupational injuries per 100,000
Bulgaria	187 (2005) ▼▼	5.8 (2005) ▼

Czech Rep.	1830 ▼▼	3.4 ▼▼

Cyprus	745 ▼	6 ▲

Estonia	561 ▲	4.3 ▲

Greece	10,684 (total/2005) ▼ 772 (2003)	5.4 (2003) ▲

Hungary	574 ▼▼	3.13 ▼▼

Latvia	177 (SLI) ▲	6.9 (2004) ▲

Lithuania	295 ▼	9.6 ▼

Poland	878 ▲	4.6 ▲

Romania	75 ▼▼	6 ▼▼

Slovakia	678 ▲	5 ▲

Slovenia	4437 ▼	3.8 ▲

Non-EU-Members		

Albania	n.s.	n.s.

Belarus	95 ▼▼	5.8 ▼

Bosnia-Herzegovina	n.s.	n.s.

Croatia	1645 ▲	5.0 ▲▲

Mazedonia	1547 (2001) ▼▼- bisection in one year	n.s.

Rep. of Moldavia	82 ▶◀	4.7 ▼▼

Montenegro	n.s	n.s.

Serbia	21924 (total/2005)	0.8 (1999/WHO)

Turkey	29 ▲	20.5 ▲▲ 13.6 (2004)

Ukraine	160 ▼▼	8.3 ▼ lowest since 1997

Trend in comparison to preceding year: ▲▲ = strong rise; ▲ = rise; ▶◀ = constant; ▼ = regressive; ▼▼ = strong regressive; n.s. = not specified

### Fatal occupational accidents

**Figure 2:**
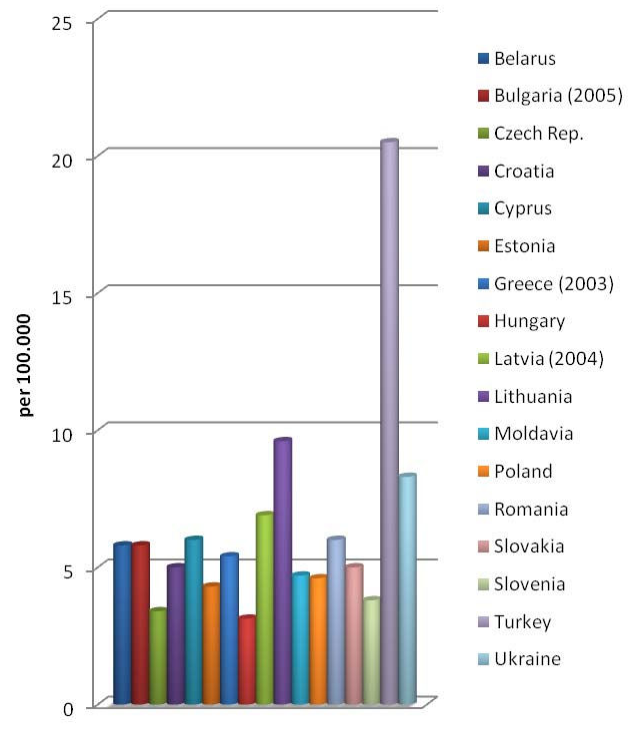
**Fatal occupational injuries 2006**. The data provided represent the number of fatal occupational injuries per 100.000 workers for the different European countries.

The situation was more heterogenous for fatal occupational accidents in 2006. Relevant data were found for 17 of the 22 countries. The highest rate was in Turkey. The rate for fatal occupational accidents was also high in Lithuania and in the Ukraine. The rates were comparatively low in Hungary, the Czech Republic and Slovenia (Figure 2). Here too there is no clear effect of EU membership on the rate of accidents, although the lowest rates were in countries which had been EU members since 2004 - i.e. two years before data collection.

In comparison to the previous year, the fatal accident rate increased in nine countries - Estonia, Greece, Croatia, Latvia, Poland, Slovakia, Slovenia, Turkey and Cyprus-, but decreased in 8 countries- Bulgaria, Lithuania, Moldavia, Romania, Czech Rep., the Ukraine, Hungary and Belarus. The rates decreased strikingly in Romania, Moldavia, the Czech Republic and Hungary. On the other hand, the rates in Croatia and Turkey increased strikingly in comparison to the previous year (Table 4). The accident rates with and without fatality increased or decreased in parallel in almost all countries. The changes in the rates were only different in Greece, Moldavia and Cyprus. An increase was found in 7 EU countries and in 3 non-EU countries, with a decrease in 4 EU countries and 4 non-EU countries. (At the time of data collection (2006), Romania was not a member of the EU and was therefore assessed as a non-member). It therefore appears that, in this context too, there is no clear link between EU membership and a decrease in accident rates. The strikingly low rates in non-EU countries may indicate that the registration system has not yet been comprehensively established.

In addition, the occupational accident rates were to be examined with respect to the different economic sectors. One reason for differences in accident rates may be that employment in different countries is dominated by different sectors, such as mining, agriculture and fishing, which may be linked to less well established occupational safety and health systems. This theory could not be tested, due to lack of differentiation of the data.

### Comparison and changes in occupational diseases

All countries have lists of officially recognised occupational diseases, even though these seem to be purely theoretical in some countries. There is however a great variation of the number and kind of diseases potentially recognized as occupational diseases -regardless of EU membership or non membership. Although the EU Commission has published an EU list of occupational diseases [1], with the recommendation that member states should adopt this, the harmonisation of the lists has not yet been implemented [2].

The Eastern European countries which are not EU members are currently often subject to fundamental reform processes, so that specific information on list systems and the number of occupational diseases are not yet available. Closed lists of occupational diseases exist in Albania, Bosnia-Herzegovina, Cyprus, Greece, Hungary, Poland, Serbia and Slovakia. Mixed systems exist in Bulgaria, the Czech Republic, Estonia, Latvia and Turkey (Table 5). The type of list system could not be established in the other 7 countries. The number of disease on the lists varies from 30 to 73: Bulgaria lists 30 (groups), Hungary 35, Slovakia 47, Greece 52 and Romania 73 (Table 4). The number of listed occupational diseases could not be established for the remaining 17 countries.

**Table 5:** Overview - Existence of lists of occupational diseases

Country	List of occupational diseases	System	Number of occupational diseases
**Albania**	yes	closed list	

**Belarus**	n.s.	n.s.	n.s.

**Bosnia-Herzegovina**	yes	closed list	n.s.

**Bulgaria**	yes	mixed system	30 groups

**Croatia**	yes	closed list	n.s.

**Cyprus**	yes	closed list	n.s.

**Czech Rep**.	yes	mixed system	n.s.

**Estonia**	yes	mixed system	n.s.

**Greece**	yes	closed list	52

**Hungary**	yes	closed list	35

**Latvia**	yes	mixed system	7 main groups with 37 subgroups

**Lithuania**	yes	n.s.	n.s.

**Macedonia**	yes	closed list	n.s.

**Rep. Moldavia**	no	n.s.	n.s.

**Montenegro**	no	n.s.	n.s.

**Poland**	yes	closed list	n.s.

**Romania**	yes	n.s.	73

**Serbia**	yes	closed list	n.s.

**Slovakia**	yes	closed list	47

**Slovenia**	yes	n.s.	n.s.

**Turkey**	yes	mixed system	n.s.

**Ukraine**	n.s.	n.s.	n.s.

n.s. not specified

**Figure 3:**
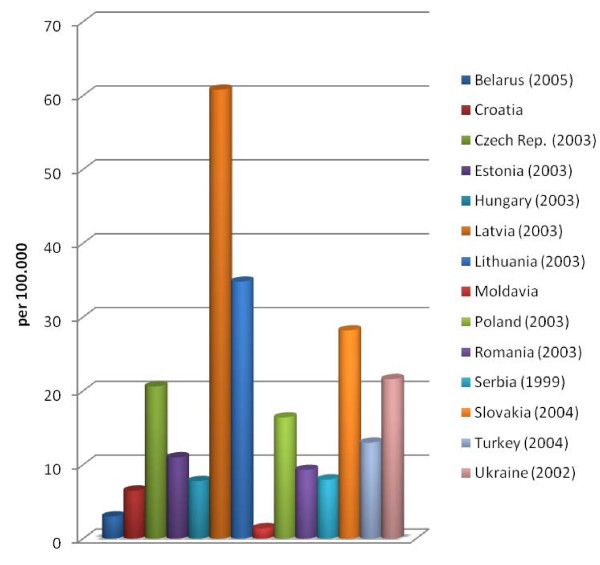
**Registered occupational diseases**. The data provided represent the number of occupational diseases per 100.000 workers for the different European countries.

It is difficult to analyse changes in registered occupational diseases (Figure 3). There are evaluable data for 14 of the 22 countries. However, some of this information does not reflect the current situation, as the data are either old or prognostic values. As most of the data are from 2004 or earlier, no conclusion can be drawn on the influence of EU membership on changes in registered occupational diseases.

The number of newly registered occupational diseases is highest in Latvia, Lithuania and Slovakia, followed by the Ukraine and the Czech Republic. The rate is particularly low in Moldavia, Belarus, Croatia, Hungary and Serbia. For 8 of the 22 countries, comparative data could be determined for the prior years. These show an increase in newly registered occupational diseases in Estonia, Slovakia and Belarus, and decreases in Latvia, Croatia, Poland, Romania and the Czech Republic. In 2004, a total of 49 occupational diseases were recorded in Slovenia. As no registration system had been established, the Labour Inspectorate stated that 1000 to 15000 newly registered occupational diseases per year could be assumed. Particularly low rates, such as in Moldavia, Belarus, Croatia or Macedonia - with currently no notified case of occupational disease - support the assumption that the official registration does not reflect the real state of affairs.

### Occupational skin diseases

**Figure 4:**
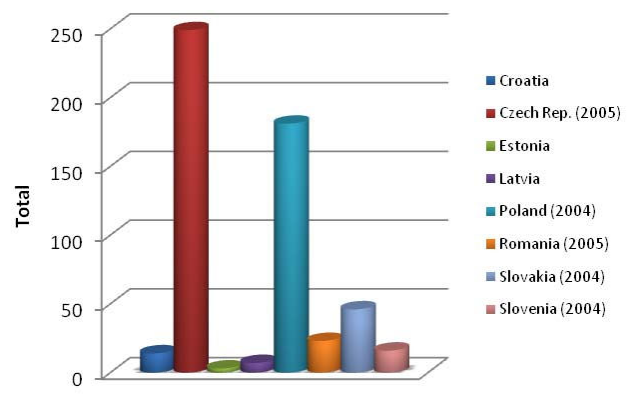
**Registered occupational skin diseases**. The data provided represent the number of registered occupational diseases per 100.000 workers for the different European countries.

Research into occupational skin diseases turned out to be particularly difficult. The data situation in non-EU states is particularly defective (Figure 4). Even correspondence with the responsible authorities in the corresponding countries (Table 1) was of little help. Relevant data could only be found in 8 of the 22 of the countries (36%). The Czech Republic and Poland currently exhibit the highest quota of registered occupational skin diseases. Slovakia is in third place. The rates in Estonia and Latvia are particularly low. On average, only two to three skin diseases are registered each year in Estonia as occupational diseases (Figure 4).

However, in comparison to previous years, it appears that the rate of newly registered skin diseases has decreased in 4 countries, i.e. in half the countries, namely Poland, Romania, Slovakia and the Czech Republic. In contrast, the number has increased in Croatia and has remained constant in Latvia. Although skin diseases are officially recognised occupational diseases in Macedonia, currently none has been registered as such. Skin diseases are the most frequent occupational diseases in Slovenia (data of 2004). In the Czech Republic and in Croatia, skin diseases are the third most frequent registered occupational disease, in Slovakia the fourth most frequent and in Latvia the sixth most frequent. In contrast, in Poland 4.8% of all registered occupational diseases are skin diseases, in Estonia about 2.6% and in Romania only 2.3%.

Occupational skin diseases could not be studied separately for the area or type of employment, as almost no differentiation had been made. However, the study performed by the "European Agency for Safety and Work" (2008) concluded for the EU-25 that: "The mining and quarrying sector shows the highest incidence rate of skin diseases (31.5), followed by manufacturing (10.4) and construction (9.1). 34% of all cases of skin diseases were registered in manufacturing, followed by construction (14%) and health and social work (9.5%) [3] (p.19)". "The occupational group of crafts and related trades workers shows the highest prevalence of skin diseases (33.2%). They are followed by the elementary occupations (22.1%), service workers, shop and market sales workers (17.8%) and plant and machine operators and assemblers (14.4%) [3]: (p.20)."

It was also impossible to examine any effect of EU membership on changes in occupational skin diseases. Of the countries for which data was available, only Croatia was not a member of the European Union. Moreover, current data could only be determined for Estonia, Latvia and the Czech Republic. All other data were from the years before entry into the EU.

## Discussion

The present study is the first comparative compilation of the social insurance systems and an analysis of the changes in occupational accidents and occupational diseases in 22 countries in Eastern Europe- extending beyond the limits of the EU. It became evident that comparative analysis could only be fragmentary, as a consequence of heterogenous, absent or unstandardised data, as well as the different degrees to which the social security system and the registration procedure had been established. Other studies have been faced with similar difficulties: "The different occupational health systems and legislations in the countries across Europe make it difficult for one to sketch a detailed picture for the whole continent" [4]. In particular, data were pause in those countries which are not members of the European Union and in which the political and state reforms and the coupled changes in the insurance system were started after the political upheavals, but have not yet been completed.

### Comparison of the accident insurance systems

In spite of their different structures, all social insurance systems recognise the special insurance cases of "occupational accident" and "occupational disease" - even though this recognition is sometimes rather theoretical. In 12 of the 22 countries, insurance against occupational accidents and occupational diseases is an independent component of the obligatory social security system. In nine countries, the risks of occupational accident and occupational disease are covered by health insurance in the short term, pension insurance in the long term and also partially by invalidity and dependent insurance. There are differences between EU member countries and non-EU members with respect to the establishment and functional efficiency of their social insurance systems.

### Occupational disease lists

All 22 countries in Eastern Europe officially have an occupational disease list. In 7 countries this is closed and in 3 countries this is a mixed system. The list system could not be unambiguously clarified for the remaining 12 countries. For those, it was also impossible to determine the number of occupational diseases included in the lists.

### Occupational accidents

The accident rates were decreasing in more than half the countries (11 of 18). On the other hand, fatal accidents increased in 9 of 17 countries. EU membership had no clear effect on the decrease in the accident rates. It is currently not posible to reach any conclusions about differences in specific risks at the workplace or any health and safety measures that may be necessary, as fatal and non-fatal occupational accidents were often not differentiated by area of employment.

### Occupational diseases

The number of newly registered occupational diseases was decreasing in more than half of the countries considered (5 of 8). As the data was too old (2004 or older), any possible effect of EU membership on this development could not be established.

### Occupational skin diseases

The data on occupational skin diseases was particularly defective. For this reason, it was not possible to consider any effect of EU membership on the development of skin diseases. In comparison to preceding years, the number of occupational skin diseases was decreasing in half the countries (4 of 8). A similar experience was made in another study on occupational skin diseases in the European Union. "The statistical data on skin diseases have to be treated with caution for several reasons. Not all EU countries were included in the data collection and statistical data are only available until 2005. There is no standard definition to approach skin diseases and there are also clear indications that the number of cases and the extent of the diseases are underestimated in the EU" [3] (p. 17).

## Conclusion

The results make it clear how important it will be in future - particularly in the eastern European countries which are non-EU members - to carry out continual standardised statistical analysis and to continue with progress in the establishment of their registration and notification procedures. Once transparency has been achieved, it will be possible to analyse future developments, to assess the success of the establishment occupational safety and health regulations, or to initiate timely improvements and ultimately to allow international comparisons between systems.

Finally, it should be said that support from an international exchange of experts can make an important contribution towards the development of social securitiy and workers health protection systems in individual countries - particularly eastern European countries which are not members of the EU. In particular, special attention should be paid to the development and establishment of local accident insurers for health and safety and rehabilitation.

## Competing interests

The authors declare that they have no competing interests.

## Authors' contributions

KRH made substantial contributions to conception and design of the study. She has made substantial contributions to the research and to the analysis and interpretation of data. She drafted the article and gave final approval of the version to be published.

BK made substantial contributions to conception and design, and was involved in the critical revision of the article. He gave final approval of the version to be published.

AN made substantial contributions to the conception and design of the study, as well as to the analysis and interpretation of the data. He was involved in the critical revision of the article and gave final approval of the version to be published.

## Supplementary Material

Additional file 1 **Literature, Internet sites and Internet documents useful in the context of the study question**. A list of literature, Internet sites an documentd from the Internet is provided. Click here for file
