# Supplementary material for: Social care and changes in occupational accidents and diseases - the situation in Eastern Europe in general and for skin diseases in particular
Source: J Occup Med Toxicol. 2009 Nov 18;4:28. doi: 10.1186/1745-6673-4-28 (PMC2791759; doi:10.1186/1745-6673-4-28)
Supplement: Additional file 1 — Literature, Internet sites and Internet documents useful in the context of the study question. A list of literature, Internet sites an documentd from the Internet is provided. [file 1745-6673-4-28-S1.doc]

# **Additional file 1**

# **Literature, Internet sites and Internet documents useful in the context of the study question**

# **Useful literature**

1. Aliza M: Occupational Health and Safety in Turkey. Middle East Report, No. 161, Health and Politics Nov./Dec. 1989: 21-23.
2. Bazas T: Occupational Health Practice in Greece. *Journal of Occupational Health 2001*, 43: 165-167.
3. Bilban M: Occupational Medicine in the Slovene Area. *Journal of Occupational Health 47/2005*: 193-200.
4. Breuer J, Weinert P, Zimmer S: Erfolgsfaktoren der Einführung und Reform von Unfallversicherungssystemen [Factors affecting the successful introduction and reform of accident insurance systems]. Ergebnisse einer Umfrage des Fachausschusses Unfallversicherung der IVSS [Results of a questionnaire of the accident insurance committee of the International Social Security Association]. *Die BG 2005,* *Nr. 12:* 738-743.
5. Fenclová Z, Urban P Pelclová D, Lebedová J, Luká E: Occupational Diseases in Health Care Workers in the Czech Republic. *Gohnet, Winter 2005*, No. 8: 10-11.
6. International Social Security Association (IVSS)/Hellenic Institute for Occupational Health and Safety: Gesundheitsrisiken der Beschäftigten im Gesundheitswesen: eine Herausforderung für die Prävention [Health risks for health workers: a challenge for prevention]. *Tagungsband des Internationalen Kolloquiums,* Athen 2007. http://www.elinyae.gr
7. Kranig A: Vergleichende Studien zu Berufskrankheiten in Europa [Comparative studies on occupational diseases in Europe]*.* *Die BG 2005*, *Nr. 12:* 760-766.
8. Pabst B: Unfallversicherungssysteme in den neuen EU-Mitgliedstaaten im Überblick [An overview of accident insurance systems in the new EU member countries]. *Die BG 2005,* *Nr. 12*: 744-748.
9. Pabst B: Polen: Arbeitsunfälle und Berufskrankheiten 2002 [Poland: Occupational accidents and occupational diseases]*. Die BG 2005*, *Nr.7*: 389.
10. Pelclova D, Fenclova Z, Lebedova J: Occupational diseases in the Czech Republic in the Year 1998. *Central European journal of public health. Czech Medical Association,* J. E. Purkyně, Prague 2000, vol. 8, No.1: 49-52.
11. Pournaras S, Tsakris A, Mandraveli K, Faitatzidiou A, Douboyas J, Tourkantonis A: Reported needlestick and sharp injuries among health care workers in a Greek general hospital*.*  *Occup. Med. Vol. 49, No. 7, 1999*: 423-426.
12. Stellman Mager J: Encyclopaedia of Occupational Health and Safety. *International Labour Organization*, 1998.
13. Zimmer S: Internationale Beziehungen [International relations]*.*  *Die BG 2005, Nr. 12*: 735-736.

# **Useful Internet sites**

- Federal Foreign Office: [http://www.auswaertiges-amt.de](http://www.auswaertiges-amt.de/)

# Committee for the European Regions: [**http://www.cor.europa.eu/de/index.htm**](http://www.cor.europa.eu/de/index.htm)

- Baltic Sea Network on Occupational Health and Safety:[http://www.balticseaosh.net](http://www.balticseaosh.net/)
- CIA World Factbook: <https://www.cia.gov/cia/publications/factbook/index.html>
- ENEPRI: The European Network of Economic Policy Research Institutes: <http://www.enepri.org/>
- European Agency for Safety and Health at Work: [http://de.osha.europa.eu](http://de.osha.europa.eu/)

# Europa auf einen Blick: [**http://www.europa-auf-einen-blick.de**](http://www.europa-auf-einen-blick.de/)

- European Union: <http://europa.eu/abc/12lessons/index2_de.htm>

# Europa Digital: [**http://www.europa-digital.de/laender/est/**](http://www.europa-digital.de/laender/est/)

- EU- Info Deutschland: [http://www.eu-info.de](http://www.eu-info.de/)
- European Commission: <http://www.consilium.europa.eu/showPage.ASP?lang=de>

# European Parlament: [**http://www.europarl.europa.eu/news/public/default_de.htm**](http://www.europarl.europa.eu/news/public/default_de.htm)

- European Economic and Social Committee: <http://www.eesc.europa.eu/index_en.asp>

# FiFo Ost: [**http://www.fifoost.org**](http://www.fifoost.org/)

- European Court: <http://www.curia.europa.eu/de/index.htm>

#### ILO: International Labour Organization: [**http://www.ilo.org/**](http://www.ilo.org/)

- ILO Laborstat: [http://laborsta.ilo.org](http://laborsta.ilo.org/)
- ISSA: SSW: Social Security Worlwide. Datenbank: http://www.ssw.issa.int

# Montenegrin Investment Promotion Agency: http://www.mipa.cg.yu/aktiv.asp

# National Institute of Statistics Romania: [**http://www.insse.ro/index_eng.htm**](http://www.insse.ro/index_eng.htm)

- Nofer Institute of Occupational Medicine Poland: http://www.imp.lodz.pl
- ONYC: General Administration of Hungarian Pension Insurance: [http://www.onyc.hu](http://www.onyc.hu/)

# European Council: [**http://www.consilium.europa.eu/showPage.ASP?lang=de**](http://www.consilium.europa.eu/showPage.ASP?lang=de)

#### World Health Organization: [**http://www.who.int**](http://www.who.int/)

#### ZUS Polish Social Insurance Institute: http://www.zus.pl

# **Useful documents in the Internet**

- Polish Embassy: Social Insurance System and Health Protection in Poland. General Information. 2004. [http://www.wirtschaft-polen.de](http://www.wirtschaft-polen.de/)
- European Foundation for the Improvement of Living and Working Conditions: Working conditions in the acceding and candidate countries. Dublin 2003.

<http://www.eurofound.europa.eu/pubdocs/2003/06/en/1/ef0306en.pdf>

- European Social Statistics: Arbeitsunfälle und arbeitsbedingte Gesundheitsbeschwerden [Occupational Accidents and Occupational Disease]. 1994-2000. European Communities 2002

http://194.95.119.6/downloads/publ/de3_arbeitsunfall.pdf

- Central Statistic Office Poland: Occupational diseases in 2000 by Voivodships.<http://www.stat.gov.pl/english/dane_spol-gosp/warunki_zycia/zdrowie/disease_treatment_by_voivodships/2000/azawod.xls>
- Central Statistic Office Poland: Occupational diseases in 2002 by Voivodships.http://www.stat.gov.pl/english/dane_spol-gosp/warunki_zycia/zdrowie/disease_treatment_by_voivodships/2002/azawod.xls
- Central Statistic Office Poland: Occupational diseases in 2003 by Voivodships.<http://www.stat.gov.pl/english/dane_spol-gosp/warunki_zycia/zdrowie/disease_treatment_by_voivodships/2003/azawod.xls>
- Central Statistic Office Poland: Occupational diseases in 1995-2002

<http://www.stat.gov.pl/english/dane_spol-gosp/warunki_zycia/zdrowie/ocup.xls>

# Czech Health Statistic 2005

[http://www.uzis.cz/download.php?ctg=10&search_name=Czech%20health%20statistics%20yearbook&region=100&mnu_id=5300](http://www.uzis.cz/download.php?ctg=10&search_name=Czech health statistics yearbook&region=100&mnu_id=5300)

- ENEPRI: Health Status and Health care systems in central and eastern European countries. Bulgaria, Estonia, Poland, Slovakia and Hungary.

<http://www.enepri.org/AHEAD/WPII/ENEPRI-WPII-comparative.pdf>

- ENEPRI: Health and Morbidity in the accession countries: Country report Bulgaria 2006 <http://www.enepri.org/AHEAD/WPII/ENEPRI-BG.pdf>
- ENEPRI: Health and Morbidity in the accession countries. Country report Poland 2006. <http://www.enepri.org/AHEAD/WPII/ENEPRI-PL.pdf>
- ENEPRI: Health and Morbidity in the Accession countries. Country Report Slovak Republic<http://www.enepri.org/AHEAD/WPII/ENEPRI-SK.pdf>

#### European Council: Albanian Insurance System

http://www.coe.int/t/dg3/sisp%5CSource%5CCRepAnn2AlbEN.PDF

- European Council: Report on the state and future of social security in Serbia and Montenegro 2005.

[http://www.coe.int/t/dg3/sisp%5CSource%5CCRepSerbMonEN.PDF](http://www.coe.int/t/dg3/sisp\Source\CRepSerbMonEN.PDF)

- Institute of Health Information and Statistics of the Czech Republic: Occupational diseases 2000-2005.

<http://www.uzis.cz/download.php?ctg=10&search_name=Occupational&region=100&kind=1&mnu_id=5300>

- Kalovska, Tanja: Macedonia. For reporting on the present state and future of social security in the countries participating in the SISP.

<http://www.coe.int/t/dg3/sisp/Source/CrepAnn1MacEn.PDF>

KOM (2005) 562: Analytical Report for the Statement on the Application of the Former Yugoslavian Republic of Macedonia for Membership of the EU. <http://ec.europa.eu/enlargement/archives/pdf/key_documents/2005/package/sec_1425_final_analytical_report_mk_de.pdf>

- KOM (2005) 534 of 25.10.2005: Report of the Committee of the European Union. Comprehensive Monitoring Report of the European Commission on the Status of the Preparation of Bulgaria and Romania for Membership

<http://eur-lex.europa.eu/LexUriServ/site/de/com/2005/com2005_0534de01.pdf>

- KOM (2004) 657 of 06.10.2004: Report of the Committee of the European Union. Comprehensive Monitoring Report of the European Commission on the Status of the Preparation of Bulgaria and Romania for Membership.

<http://eur-lex.europa.eu/LexUriServ/site/de/com/2004/com2004_0657de01.pdf>

- KOM (2003) 676 of 05.11.2003: Continuation of the Expansion. Strategy Paper Report from the European Committee on the Progress of Bulgaria, Romania and Turkey towards Membership.

<http://eur-lex.europa.eu/LexUriServ/site/de/com/2003/com2003_0676de01.pdf>

- KOM (2002) 700 of 9.10.2002: Strategy Paper and Report of the European Committee on the Progress of each Applicant towards Membership.

<http://eur-lex.europa.eu/LexUriServ/site/de/com/2002/com2002_0700de01.pdf>

- Commission of the European Union: Recommendation of the Committee mpfehlung of 19 September 2003 on the European List of Occupational Diseases. *Amtsblatt der Europäischen Union of 25. September 2003:*28-34
- Ministry of Labour and Social Solidarity Romania: Overview of the Romanian Social Security System 2003.

http://www.ssk.gov.tr/sskdownloads/sigorta/Romanya_MEVZUATI_english.doc

- Ministry of Welfare, Department of Labour Latvia: Occupational Safety and Health Strategy in Latvia 2002.

http://www.balticseaosh.net/latvia/Latvia_OSH_Strategy.doc

- MISEEC Tables 2002: Mutual Information System on Social Security in the mid-European and East European Countries: Bulgaria, Czech Republic, Estonia, Hungary, Latvia, Lithuania, Poland, Romania, Slovakia and Slovenia. Situation on 1 January 2002.
- I: Financing <http://ec.europa.eu/employment_social/missceec/table1_en.pdf>
- III. Illness <http://ec.europa.eu/employment_social/missceec/table3_en.pdf>
- V: Invalidity <http://ec.europa.eu/employment_social/missceec/table5_en.pdf>
- VIII: Occupational Accidents and Occupational Disease

<http://ec.europa.eu/employment_social/missceec/table8_en.pdf>

- MISSOC-INFO 02/2004: Social Security in the 10 New Member States: Estonia.

<http://ec.europa.eu/employment_social/missoc/2004/022004/et_de.pdf>

- MISSOC-INFO 02/2004: Social Security in the 10 New Member States: Latvia.

<http://ec.europa.eu/employment_social/missoc/2004/022004/lv_de.pdf>

- MISSOC Info 02/2004: Social Security in the 10 New Member States: Lithuania.

<http://ec.europa.eu/employment_social/missoc/2004/022004/lt_de.pdf>

- MISSOC-INFO 02/2004: Social Security in the 10 New Member States: Poland

<http://ec.europa.eu/employment_social/missoc/2004/022004/pl_de.pdf>

- MISSOC-INFO 02/2004: Social Security in the 10 New Member States: Slovenia.

<http://ec.europa.eu/employment_social/missoc/2004/022004/si_de.pdf>

- MISSOC Info 02/2004: Social Security in the 10 New Member States: Czech Republic.

<http://ec.europa.eu/employment_social/missoc/2004/022004/cs_de.pdf>

### MISSOC: Country Information on the Development of Social Security

**[http://ec.europa.eu/employment_social/social_protection/missoc_info_de.htm#01/2005](http://ec.europa.eu/employment_social/social_protection/missoc_info_de.htm#01/2005)**

- MISSOC: Study on the social protection Systems in the 13 applicant Countries. Poland country study.

<http://ec.europa.eu/employment_social/social_protection/docs/poland_final.pdf>

- MISSOC Vergleichende Tabellen, PART 1: Social Security in the Member States of the European Union, in the European Economic Area and in Switzerland. Part 1: Belgium, Bulgaria, Czech Republic, Denmark. 1 January 2007

<http://ec.europa.eu/employment_social/missoc/2007/tables_part_1_de.pdf>

- MISSOC Comparative Tables, PART 2: Social Security in the Member States of the European Union, in the European Economic Area and in Switzerland. Part 2: Germany, Estonia, Greece, Spain. 1 January 2007

http://ec.europa.eu/employment_social/missoc/2007/tables_part_2_de.pdf

- MISSOC Comparative Tables, PART 4: Social Security in the Member States of the European Union, in the European Economic Area and in Switzerland. Part 4: Cyprus, Latvia, Leichtenstein, Lithuania. 1 January 2007

<http://ec.europa.eu/employment_social/missoc/2007/tables_part_4_de.pdf>

- MISSOC Comparative Tables, PART 5: Social Security in the Member States of the European Union, in the European Economic Area and in Switzerland. Part 5: Luxembourg, Hungary, Malta, Netherlands. 1 January 2007

<http://ec.europa.eu/employment_social/missoc/2007/tables_part_5_de.pdf>

- MISSOC Comparative Tables, PART 6: Social Security in the Member States of the European Union, in the European Economic Area and in Switzerland. Part 6: Norway, Austria, Poland, Portugal. 1 January 2007

<http://ec.europa.eu/employment_social/missoc/2007/tables_part_6_de.pdf>

- MISSOC Comparative Tables, PART 7: Social Security in the Member States of the European Union, in the European Economic Area and in Switzerland. 1 January 2007

<http://ec.europa.eu/employment_social/missoc/2007/tables_part_7_de.pdf>

- Nika, Milos: Serbia and Montenegro: Report on the present state and future of Social Security.

<http://www.coe.int/t/dg3/sisp/Source/CRepSerbMonEN.PDF>

## Obarčanin, Jasmina: Report on the present state and future of Social Security in Bosnia-Herzegovina.

# [**http://www.coe.int/t/dg3/sisp%5CSource%5CCRepAnn1BihEN.PDF**](http://www.coe.int/t/dg3/sisp\Source\CRepAnn1BihEN.PDF)

- Olmezoglu, Zeynep: Occupational Health and Safety in Turkey. Lecture on 2 October 2005, Eretria, Greece [http://www.euroipn.org/cerepri/pre_event/files/presentations/Workshops/WS%2007%20Zeynep%20Olmezoglu.pdf](http://www.euroipn.org/cerepri/pre_event/files/presentations/Workshops/WS 07 Zeynep Olmezoglu.pdf)

# European Council: Presse 330 No. 15487/06 – 2767. Meeting of the Council for Employment, Social Politics, Health and Consumer Protection. Brussels 1.12.2006

<http://www.consilium.europa.eu/ueDocs/cms_Data/docs/pressData/de/lsa/92353.pdf>

- Rismondo, Mihovil: Croatia: For reporting on the present state and future of social security in the countries participating in the SISP.

<http://www.coe.int/t/dg3/sisp/Source/CRepAnn1CroEN.PDF>

- Selita, Mirela: Albania: For reporting on the present state and future of social security in the countries participating in the SISP.

<http://www.coe.int/t/dg3/sisp/Source/CRepAnn1AlbEN.PDF>

- State Labour Inspectorate of the Republic of Lithuania: Annual Report of the Labour Inspectorate: Year 2005. <http://www.vdi.lt/index.php?-757119335>

# Statistical Yearbook of Serbia 2006:

# [**http://webrzs.statserb.sr.gov.yu/axd/en/god.htm**](http://webrzs.statserb.sr.gov.yu/axd/en/god.htm)

- U.S. Social Security Administration : Social Security Programs throughout the World: Europe 2006.

http://www.ssa.gov/policy/docs/progdesc/ssptw/2006-2007/europe/guide.html

# WHO: Highlights on Health in Belarus 2000.

http://www.euro.who.int/document/e72016.pdf

# WHO: Highlights on Health in Bulgaria. [**http://www.euro.who.int/eprise/main/WHO/Progs/CHHBUL/sum/20041125_24?language=German**](http://www.euro.who.int/eprise/main/WHO/Progs/CHHBUL/sum/20041125_24?language=German)

- WHO: Health care systems in transition – Bulgaria 2003.

<http://www.euro.who.int/document/e81760.pdf>

- WHO: Highlights on Health in Greece. 2004

<http://www.euro.who.int/highlights>

- WHO: Highlights on Health in the Republic of Moldova.

<http://www.euro.who.int/Document/e88552.pdf>

- WHO: Highlights on Health in Poland. 2005

<http://www.euro.who.int/document/E88745.pdf>

## WHO: Highlights on Health Romania.

<http://ec.europa.eu/health/ph_projects/1999/monitoring/romania_en.pdf>

# WHO: Highlights on Health Slovakia.

<http://www.euro.who.int/Document/e88407.pdf>

##### WHO: 10 questions about the new EU-neighbours – Belarus 2006.

http://www.euro.who.int/document/e88202_belarus.pdf

# WHO: 10 questions about the ten new EU neighbours.

<http://www.euro.who.int/InformationSources/Publications/Catalogue/20060301_2>

- WHO: 10 questions about the new EU-neighbours – Moldova 2006.

http://www.euro.who.int/document/e88202_moldova.pdf

# WHO: 10 questions about health: Romanian.

http://www.euro.who.int/Document/E88202_Romania.pdf

# WHO: 10 questions about health: Serbia and Montenegro.

<http://www.euro.who.int/Document/E88202_Serbia.pdf>

- ZUS: Social Insurance Institute Poland: Social Assurance in Poland. Information and Facts 2006

<http://www.zus.pl/german/deutsche.pdf>
